# Long-Term Risk of Cardiovascular Disease After Contemporary Left-Sided Breast Radiation Therapy

**DOI:** 10.1001/jamanetworkopen.2026.4098

**Published:** 2026-04-01

**Authors:** Erika Nakajima, Lena Nguyen, Ning Liu, Danielle Rodin, Eitan Amir, Peter C. Austin, Paaladinesh Thavendiranathan, Husam Abdel-Qadir

**Affiliations:** 1 Women’s College Hospital, Toronto, Ontario, Canada; 2 Temerty Faculty of Medicine, University of Toronto, Toronto, Ontario, Canada; 3 ICES (formerly known as the Institute for Clinical Evaluative Sciences), Toronto, Ontario, Canada; 4 Princess Margaret Cancer Centre, Toronto, Ontario, Canada; 5 Department of Radiation Oncology, University of Toronto, Toronto, Ontario, Canada; 6 Institute of Health Policy, Management, and Evaluation, University of Toronto, Toronto, Ontario, Canada; 7 Peter Munk Cardiac Centre, Toronto, Ontario, Canada

## Abstract

**Importance:**

External beam radiation therapy (EBRT) for left-sided breast cancer historically increased the risk of cardiovascular disease (CVD). Advances in EBRT have reduced mean heart dose, but contemporary population-level data on long-term cardiovascular outcomes remain limited.

**Objective:**

To examine whether contemporary left-sided EBRT is associated with different long-term CVD risk than right-sided EBRT among women with breast cancer.

**Design, Setting, and Participants:**

This population-based, retrospective cohort study used linked administrative health databases in Ontario, Canada, to identify women who received EBRT after a diagnosis of unilateral breast cancer between April 1, 2002, and December 31, 2017. Patients were followed up through February 28, 2025, for most outcomes and December 31, 2022, for cause-specific mortality. Data analysis was completed in August 2023 (with revisions in August 2025).

**Exposure:**

Tumor laterality (left vs right).

**Main Outcomes and Measures:**

The primary outcome was hospitalization with a most responsible diagnosis of CVD. Secondary outcomes included all-cause mortality, cardiovascular mortality, hospitalizations for specific CVD diagnoses, coronary revascularization, and new diagnoses of ischemic heart disease, heart failure, and atrial fibrillation. Cumulative incidence functions estimated the 15-year risk of outcomes accounting for the competing risk of death; event rates per 100 person-years captured recurrent events.

**Results:**

Among 76 586 women (mean [SD] age, 59 [12] years; 38 427 [50.2%] with left-sided tumors) followed up for a median (IQR) of 10.9 (7.7-15.2) years, the 15-year cumulative incidence of first CVD hospitalization did not differ by laterality (left: 13.8% [95% CI, 13.4%-14.2%]; right: 13.5% [95% CI, 13.1%-13.9%]; *P* = .43). In women without preexisting CVD, new diagnoses of heart failure (10.2% [95% CI, 9.9%-10.6%] vs 9.6% [95% CI, 9.2%-10.0%]; *P* = .01) and ischemic heart disease (13.6% [95% CI, 13.2%-14.0%] vs 12.8% [95% CI, 12.4%-13.2%]; *P* = .03) were slightly more frequent after left-sided EBRT. The rate of CVD hospitalizations when including recurrent events was modestly higher for left-sided disease (1.72 vs 1.63 per 100 person-years; *P* < .001). Among women with preexisting CVD, there were no differences in all-cause mortality or recurrent CVD hospitalizations.

**Conclusions and Relevance:**

In this cohort study of women treated with EBRT for breast cancer in the past 2 decades, left-sided breast cancer radiation therapy was associated with minimal increases in long-term cardiovascular risk. These findings suggest that contemporary photon-based EBRT techniques have substantially reduced the cardiovascular risk historically associated with left-sided breast cancer radiation therapy.

## Key Points

**Question:**

Is contemporary left-sided external beam radiation therapy (EBRT) for breast cancer associated with higher long-term risk of cardiovascular disease?

**Findings:**

In this population-based cohort study of 76 586 women treated with EBRT between 2002 and 2017, there was no difference in the incidence of first cardiovascular hospitalization between those with left- and right-sided breast cancer. Women with left-sided disease had slightly higher rates of new heart failure and ischemic heart disease diagnoses and recurrent cardiovascular hospitalizations.

**Meaning:**

This study’s results suggest that contemporary photon-based EBRT techniques have reduced the excess long-term cardiovascular risk historically associated with left-sided breast cancer radiation therapy.

## Teaser

This cohort study compares the long-term risk of cardiovascular disease between women receiving contemporary external beam radiation therapy for left- vs right-sided breast cancer.

## Introduction

External beam radiation therapy (EBRT) significantly reduces the risk of cancer recurrence and cancer-related mortality in females with early-stage breast cancer (BC).^1,2,3^ EBRT techniques used before the 1980s exposed surrounding organs to large doses of radiation, so women with left-sided tumors historically received higher mean heart doses (MHD) than women with right-sided tumors.^4,5,6,7^ Radiation to the heart increases the risk of coronary artery disease (CAD), cardiomyopathy, valvulopathy, arrhythmias, and pericardial disease.^8^ Hence, women with left-sided BC treated with EBRT in the 1970s to early 1980s had higher rates of cardiovascular disease (CVD) mortality compared with women with right-sided BC.^6,7^

Radiotherapy delivery has since been modified to reduce MHD. Two-dimensional radiation therapy was replaced with photon-based 3-dimensional conformal radiation therapy and intensity-modulated radiation therapy.^5,9,10^ Other maneuvers, including deep inspiration breath hold, were developed to move the heart away from the radiation field.^11,12^ Improvements in computed tomography simulation, evaluation of dose to organs at risk, planning software, and other machine technology have led to a downward trend in MHD from left-sided breast radiation.^13,14^

Proton beam therapy has recently emerged as a novel alterative to photon-based radiotherapy that can substantially reduce cardiac radiation exposure.^15,16,17^ The limited availability and higher cost of proton beam radiation make it important to reappraise if contemporary left-sided EBRT with photon therapy meaningfully increases CVD risk in patients with BC. Recent data suggest that the risk of CAD associated with left-sided EBRT has been substantially attenuated.^18,19,20,21^ The small sample size or shorter follow-up of prior studies^48,49^ obviated the ability to detect smaller differences in risk, and there are limited data for forms of CVD beyond CAD.

We conducted a population-based cohort study of women who received contemporary photon-based EBRT after a diagnosis of BC. The primary objective was to compare the long-term risk of CVD between women receiving radiotherapy for left- vs right-sided BC. We hypothesized that women with left-sided BC would have no significant difference in CVD risk after EBRT compared with those with right-sided BC.

## Methods

### Data Sources

This population-based, retrospective cohort study leveraged linked administrative datasets in Ontario, Canada, where all residents receive coverage for medically necessary physician care and hospital services through the Ontario Health Insurance Plan (OHIP). The OHIP database contains data on physician billing claims, including the service provided and underlying diagnosis. The Ontario Cancer Registry contains data on all patients with pathology report–confirmed BC diagnoses, including tumor laterality.^22^ The Canadian Institute of Health Information Discharge Abstract Database contains data on hospitalized patients, including their medical diagnoses, whereas the National Ambulatory Care Reporting System contains data on hospital-based ambulatory care (including chemotherapy and radiation therapy) as well as emergency department visits. The Same Day Surgery dataset contains information on same day surgeries. The Cancer Activity Level Reporting Radiation dataset records details on radiation therapy plans and chemotherapy exposures at regional cancer centers. These datasets were linked using unique encoded identifiers and analyzed at ICES (formerly the Institute for Clinical Evaluative Sciences),^23^ an independent, nonprofit research institute whose legal status under Ontario’s health information privacy law allows it to collect and analyze health care and demographic data, without consent, for health system evaluation and improvement. The use of the data in this project is authorized under §45 of Ontario’s Personal Health Information Protection Act and does not require review by a research ethics board or patient informed consent.^24^ Methods of this study have been developed and reported in concordance with Strengthening the Reporting of Observational Studies in Epidemiology (STROBE) guidelines for cohort studies.

### Cohort Creation

Using these datasets, we created a cohort of patients diagnosed with BC who underwent EBRT in Ontario between April 1, 2002, and December 31, 2017. Inclusion was limited to patients who received their first radiation treatment within 2 years after initial BC diagnosis. All patients included in our cohort received photon-based therapy because proton therapy is not currently being used in Ontario. The index date was that of first documented radiation treatment. Exclusion criteria included non-Ontario residence, male sex, OHIP eligibility for less than 2 years before first radiation treatment, invalid death dates, initial presentation with metastatic disease (stage IV) or ductal carcinoma in situ (stage 0), prior malignant tumor, bilateral BC at time of diagnosis, unknown tumor laterality, and second cancer diagnosis before first radiation treatment. The key exposure was having a left-sided breast tumor, with the comparator group being women with right-sided breast tumors. Cancer laterality was determined from the Ontario Cancer Registry and served as a natural experiment because tumor side is quasi-random with respect to baseline cardiovascular risk.

The primary outcome was a composite of hospitalization with a most responsible diagnosis of CVD (*International Statistical Classification of Diseases and Related Health Problems, Tenth Revision [ICD-10]* codes I00-I78). Secondary outcomes included all-cause mortality, cardiovascular mortality, and hospitalizations with the following most responsible diagnoses (in separate analyses): acute myocardial infarction (AMI; *ICD-10* codes I21 and I22), stroke (*ICD-10* codes I60, I61, I63 [excluding I63.6], I64, and H34.1), heart failure (HF; *ICD-10* code I50), and pericardial diseases (*ICD-10* codes I30-I32). We also reported rates of coronary revascularization with percutaneous coronary intervention (*Canadian Classification of Diagnostic, Therapeutic, and Surgical Procedures [CCP]* codes 4802 and 4803; *Canadian Classification of Health Interventions [CCI]* codes 1IJ50, 1IJ54, and 1IJ57GQ) or coronary artery bypass graft surgery (*CCP* code 481; *CCI* code 1IJ76). Additional composite outcomes included a major adverse cardiovascular event composite outcome (AMI, stroke, or cardiovascular death) and a cardiac-specific composite outcome (AMI, HF, pericardial disease, valvular disease, or arrhythmia-related hospitalization).

Furthermore, we conducted 2 subgroup analyses: (1) women without preexisting CVD (ie, excluding prior AMI, stroke, HF, atrial fibrillation, or valvular disease) to analyze new diagnoses of ischemic heart disease (IHD), HF, or AF and (2) women with preexisting CVD because the difference in risk may be accentuated in this subgroup. Further details on the case definitions for incident diagnoses can be found in the eMethods in Supplement 1. The date of last follow-up was the earliest of the development of the outcome of interest, new cancer diagnosis date, death date, or that of last available data (February 28, 2025, for most outcomes and December 31, 2022, for cause-specific mortality).

### Statistical Analysis

Patients were stratified by tumor laterality (left vs right) for baseline comparisons. Continuous variables were summarized using the mean (SD), and differences between the groups were compared using the independent-sample *t* test. Categorical variables were summarized using numbers (percentages) and were compared using the χ^2^ test. Given our large sample size, we relied on standardized differences rather than *P* values to assess the relevance of baseline differences between groups but present both metrics to readers.

We used cause-specific hazards regression to study the association of tumor laterality with outcomes. We used the Aalen-Johansen estimate of the cumulative incidence function to estimate the risk of outcomes during the 15-year period after first radiation treatment, while accounting for the competing risk of death. To account for recurrent events, we calculated the event rate per 100 person-years with 95% CIs. We also used the Andersen-Gill approach to quantify the association of tumor laterality with the hazard of hospitalization for any CVD, including recurrent events. Given that we expected tumor laterality to be quasi-randomized, we did not plan to conduct multivariable regression unless there were important differences in baseline cardiovascular risk factors by tumor laterality. At the request of peer reviewers, we conducted sensitivity analyses in which the following covariates were added to the regression models: age, year of radiation treatment, diabetes, hypertension, surgery type (mastectomy vs breast-conserving), and receipt of chemotherapy.

To assess for the consistency of the association of tumor laterality with the primary outcome, we tested for the presence of a statistically significant interaction between tumor laterality and each the following variables (in separate analyses): age, treatment era (operationalized as a 3-level categorical variable: 2002-2007, 2008-2012, and 2013-2017), receipt of chemotherapy, receipt of trastuzumab, history of diabetes or hypertension, and prior CVD. For patients treated after 2008, we also tested the significance of an interaction between breast cancer laterality and (1) stage and (2) surgery type (mastectomy or breast-conserving surgery). The analysis assessing the interaction with treatment era was censored at 10-year follow-up. If the interaction term was statistically significant, we repeated the analyses after stratifying the cohort on the given variable. The stratified analysis by prior CVD was planned a priori, whereas the other stratified analyses were conducted post hoc at the request of peer reviewers.

All analyses were performed using SAS Enterprise Guide 7.1 (SAS Institute Inc). For baseline characteristics, differences were considered clinically relevant if standardized differences were greater than 0.1.^25^ The statistical significance of outcome comparisons between groups was defined as a 2-tailed *P* < .05. Cells with fewer than 6 individuals are suppressed to reduce the risk of reidentification as per ICES contractual obligations with data providers.

## Results

### Patient Characteristics

**Figure 1.**
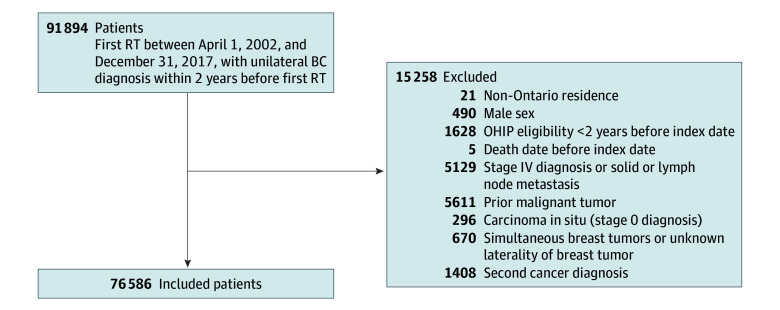
Flow Diagram of Cohort Selection BC indicates breast cancer; OHIP, Ontario Health Insurance Plan; RT, radiation therapy.

We identified 76 586 women eligible for the study (Figure 1), of whom 38 427 (50.2%) had left-sided breast tumors. The baseline characteristics of the cohort, stratified by tumor laterality, are presented in Table 1. The mean (SD) age was 59 (12) years. There were no differences in any measured baseline characteristics between the left- and right-sided tumor cohorts, with all standardized differences for measured baseline variables between left- and right-sided BC being 0.02 or less. Baseline characteristics for subgroups with and without prior CVD are presented in eTables 1 and 2 in Supplement 1.

**Table 1.** Baseline Characteristics by Tumor Laterality

Characteristic	No. (%) of patients^a^			Standard difference	*P* value
	Total (N = 76 586)	Left (n = 38 427)	Right (n = 38 159)		
Age at first RT, mean (SD), y	59.2 (12.2)	59.3 (12.2)	59.2 (12.2)	0.01	.22
Follow-up time, median (IQR), y	10.9 (7.7-15.2)	10.9 (7.7-15.2)	10.9 (7.7-15.2)	0.01	.40
Reason for censoring					
Death	17 654 (23.1)	8917 (23.2)	8737 (22.9)	0.01	.58
Second cancer diagnosis	11 071 (14.5)	5533 (14.4)	5538 (14.5)	<0.01	
End of study period	47 861 (62.5)	23 977 (62.4)	23 884 (62.6)	<0.01	
Year of first RT					
2002	4215 (5.5)	2110 (5.5)	2105 (5.5)	<0.01	.40
2003	3946 (5.2)	2001 (5.2)	1945 (5.1)	0.01	
2004	3996 (5.2)	2056 (5.4)	1940 (5.1)	0.01	
2005	4446 (5.8)	2220 (5.8)	2226 (5.8)	<0.01	
2006	4538 (5.9)	2216 (5.8)	2322 (6.1)	0.01	
2007	4410 (5.8)	2256 (5.9)	2154 (5.6)	0.01	
2008	4579 (6.0)	2328 (6.1)	2251 (5.9)	0.01	
2009	4721 (6.2)	2325 (6.1)	2396 (6.3)	0.01	
2010	4921 (6.4)	2488 (6.5)	2433 (6.4)	<0.01	
2011	5201 (6.8)	2611 (6.8)	2590 (6.8)	<0.01	
2012	5278 (6.9)	2624 (6.8)	2654 (7.0)	0.01	
2013	5299 (6.9)	2657 (6.9)	2642 (6.9)	<0.01	
2014	5530 (7.2)	2721 (7.1)	2809 (7.4)	0.01	
2015	5484 (7.2)	2768 (7.2)	2716 (7.1)	<0.01	
2016	5714 (7.5)	2912 (7.6)	2802 (7.3)	0.01	
2017	4308 (5.6)	2134 (5.6)	2174 (5.7)	0.01	
Neighborhood income quintile					
First	12 755 (16.7)	6498 (16.9)	6257 (16.4)	0.01	.20
Second	14 643 (19.1)	7344 (19.1)	7299 (19.1)	<0.01	
Third	14 978 (19.6)	7447 (19.4)	7531 (19.7)	0.01	
Fourth	16 169 (21.1)	8178 (21.3)	7991 (20.9)	0.01	
Fifth	17 872 (23.3)	8875 (23.1)	8997 (23.6)	0.01	
Rural residence	5835 (7.6)	2854 (7.4)	2981 (7.8)	0.02	.13
Neighborhood racialized and newcomer populations quintile					
First	14 155 (18.5)	7083 (18.4)	7072 (18.5)	<0.01	.33
Second	14 614 (19.1)	7260 (18.9)	7354 (19.3)	0.01	
Third	15 136 (19.8)	7534 (19.6)	7602 (19.9)	0.01	
Fourth	15 501 (20.2)	7864 (20.5)	7637 (20.0)	0.01	
Fifth	16 526 (21.6)	8348 (21.7)	8178 (21.4)	0.01	
Stage					
I	24 013 (31.4)	11 980 (31.2)	12 033 (31.5)	0.01	.40
II	20 801 (27.2)	10 531 (27.4)	10 270 (26.9)	0.01	
III	8781 (11.5)	4423 (11.5)	4358 (11.4)	<0.01	
Unknown	22 991 (30.0)	11 493 (29.9)	11 498 (30.1)	0.01	
Surgery type					
Breast-conserving surgery	57 560 (75.2)	28 885 (75.2)	28 675 (75.1)	<0.01	.36
Partial mastectomy	233 (0.3)	120 (0.3)	113 (0.3)	<0.01	
Mastectomy	13 823 (18.0)	6983 (18.2)	6840 (17.9)	0.01	
None	4970 (6.5)	2439 (6.3)	2531 (6.6)	0.01	
Chemotherapy	42 309 (55.2)	21 397 (55.7)	20 912 (54.8)	0.02	.01
Trastuzumab	7079 (9.2)	3637 (9.5)	3442 (9.0)	0.02	.03
Time from diagnosis to RT, mean (SD), d	165.86 (84.03)	166.58 (84.15)	165.14 (83.91)	0.02	.02
Diabetes	10 448 (13.6)	5286 (13.8)	5162 (13.5)	0.01	.36
Hypertension	32 600 (42.6)	16 437 (42.8)	16 163 (42.4)	0.01	.24
Chronic obstructive pulmonary disorder	2787 (3.6)	1381 (3.6)	1406 (3.7)	0.01	.50
Chronic kidney disease	1465 (1.9)	734 (1.9)	731 (1.9)	<0.01	.96
Acute myocardial infarction	551 (0.7)	279 (0.7)	272 (0.7)	<0.01	.83
Hospitalization for stroke	310 (0.4)	147 (0.4)	163 (0.4)	0.01	.33
Heart failure	1930 (2.5)	971 (2.5)	959 (2.5)	<0.01	.90
Ischemic heart disease	8660 (11.3)	4370 (11.4)	4290 (11.2)	<0.01	.57
Atrial fibrillation	2991 (3.9)	1468 (3.8)	1523 (4.0)	0.01	.22
Valvular disease	370 (0.5)	200 (0.5)	170 (0.4)	0.01	.13

Abbreviation: RT, radiation therapy.

^a^ Unless otherwise indicated.

### Outcomes

During a median (IQR) follow-up period of 10.9 (7.7-15.2) years, there were a total of 14 370 hospitalizations for CVD (16.7%), 8432 hospitalizations for HF (9.8%), 2575 hospitalizations for AMI (3.0%), 2444 hospitalizations for stroke (2.9%), and 652 hospitalizations for pericardial disease (0.8%). In the subgroup of 71 675 individuals without CVD before first radiation treatment, there were 8368 new diagnoses of IHD (11.7%), 6013 diagnoses of AF (8.4%), and 6204 diagnoses of HF (8.7%). A total of 17 654 patients (23.1%) died, with no difference in all-cause mortality at 15 years between patients with left-sided (26.1%; 95% CI, 25.5%-26.6%) and right-sided (26.0%; 95% CI, 25.5%-26.5%) tumors (*P* = .43).

### Risk of First Cardiovascular Event

**Figure 2.**
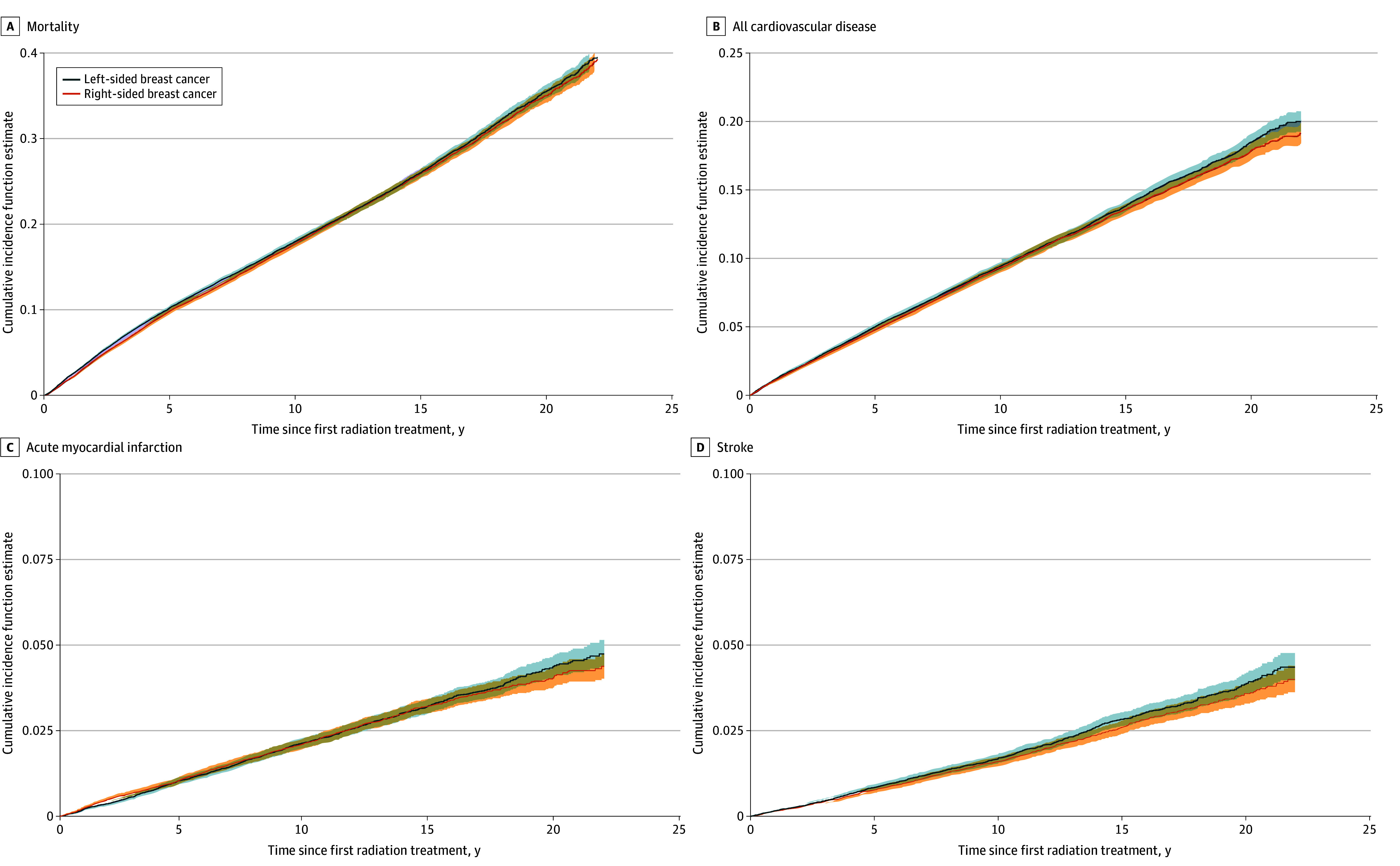
Cumulative Incidence Function Curves of All-Cause Mortality, First Hospitalization Cardiovascular Disease, Acute Myocardial Infarction, and Stroke Shaded areas indicate 95% CIs.

Figure 2 illustrates the cumulative incidence of hospitalization for any CVD, selected cardiovascular outcomes, and all-cause mortality up to 15 years after first radiation treatment. There were no differences in the 15-year incidence of first hospitalization for any CVD (left: 13.8%; 95% CI, 13.4%-14.2%; right: 13.5%; 95% CI, 13.1%-13.9%; *P* = .43). After adjusting for baseline covariates, tumor laterality was not significantly associated with the hazard of hospitalization for CVD (hazard ratio, 1.02; 95% CI, 0.98-1.06; *P* = .36) (eTable 3 in Supplement 1). Among secondary outcomes, there was no difference in the incidence of AMI (left: 3.2%; 95% CI, 3.0%-3.5%; right: 3.2%; 95% CI, 3.0%-3.5%; *P* = .93) or stroke (left: 2.8%; 95% CI, 2.6%-3.0%; right: 2.6%; 95% CI, 2.4%-2.8%; *P* = .14).

The eFigure in Supplement 1 presents cumulative incidence function curves for the remaining cardiovascular outcomes. There was no significant difference in the incidence of pericardial disease during the 15-year follow-up (left: 0.8%; 95% CI, 0.7%-0.9%; right: 0.8%; 95% CI, 0.7%-0.9%; *P* = .20). When we assessed shorter intervals in the early post–radiation therapy period (0-2 years, 2-5 years, 5-10 years, and >10 years), we observed a significant difference between patients with left- and right-sided tumors in years 0 to 2 after radiation therapy (left: 0.2%; 95% CI, 0.2%-0.3%; right: 0.1%; 95% CI, 0.1%-0.1%; *P* < .001). There was no difference in the risk of pericardial disease at any of the other intervals after radiation therapy (eTable 4 in Supplement 1). There was no difference in the 15-year incidence of coronary artery revascularization between patients with left-sided (2.2%; 95% CI, 2.1%-2.4%) and right-sided (2.2%; 95% CI, 2.0%-2.4%) tumors (*P* = .97). We also did not observe any significant differences in the incidence of the major adverse cardiovascular event composite outcome or cardiac-specific composite outcome between women with left- and right-sided breast tumors up to 15 years after radiation treatment. A summary of cumulative incidences for each outcome at 5, 10, and 15 years after radiation therapy is presented in eTable 5 in Supplement 1. When limiting our analyses to the subgroup with preexisting CVD, we observed similar patterns wherein there was there was no difference in all-cause mortality or first hospitalization for any major CVD, AMI, stroke, or HF between left- vs right-sided EBRT (eTable 6 in Supplement 1).

**Figure 3.**
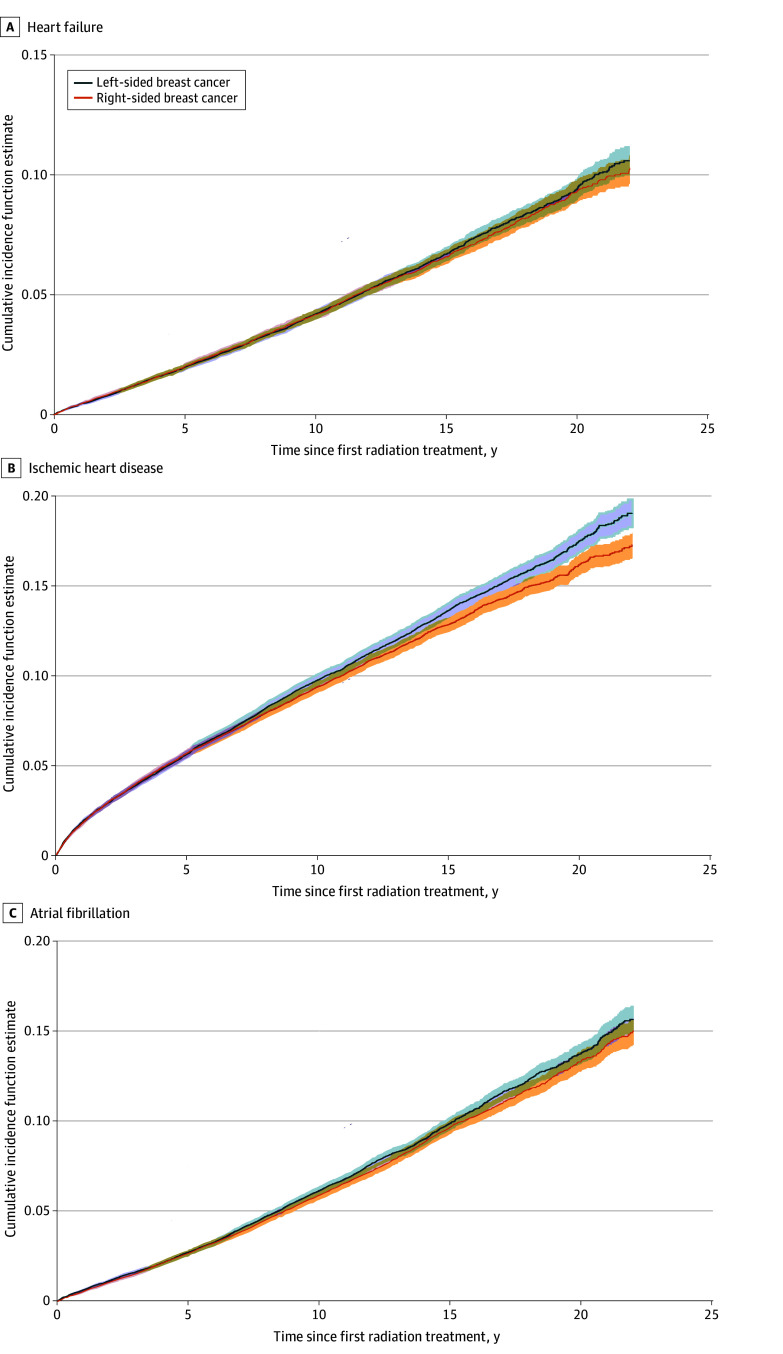
Cumulative Incidence Function Curves of New Diagnoses of Heart Failure, Ischemic Heart Disease, and Atrial Fibrillation Shaded areas indicate 95% CIs.

Figure 3 demonstrates the cumulative incidence function curves for new diagnoses of HF, IHD, and AF in the subset of the cohort that was free from CVD at baseline. The incidence of HF at 15 years in patients with left-sided tumors (10.2%; 95% CI, 9.9%-10.6%) was significantly higher than in patients with right-sided tumors (9.6%; 95% CI, 9.2%-10.0%; *P* = .01 for comparison between groups). Patients with left-sided tumors also had a significantly increased risk of new diagnoses of IHD at 15 years after radiation therapy compared with patients with right-sided tumors (left: 13.6%; 95% CI, 13.2%-14.0%; right: 12.8%; 95% CI, 12.4%-13.2%; *P* = .03). There was no significant difference between the 2 groups in the incidence of new diagnoses of atrial fibrillation (left: 9.9%; 95% CI, 9.5%-10.3%; right: 9.7%; 95% CI, 9.3%-10.1%; *P* = .26).

Furthermore, there was no significant interaction between laterality and the following variables: treatment era, history of diabetes or hypertension, prior CVD, stage, surgery type, or receipt of trastuzumab. There was a significant interaction between laterality and age (*P* for interaction = .03) and receipt of chemotherapy (*P* for interaction = .01). Among women younger than 50 years, left-sided tumor laterality was significantly associated with increased risk of CVD at 15 years (left: 4.8%; 95% CI, 4.3%-5.3%; right: 3.9%; 95% CI, 3.4%-4.3%; *P* = .02), whereas there was no significant difference by laterality among women 50 years or older (eTable 7 in Supplement 1). Among women who received chemotherapy, left-sided tumor laterality was associated with an increased risk of CVD at 15 years (left: 11.3%; 95% CI, 10.8%-11.9%; right: 10.3%; 95% CI, 9.8%-10.8%; *P* = .01), whereas there was no significant difference by laterality in women who did not receive chemotherapy (eTable 8 in Supplement 1).

### Rate of CVD

The rates of cardiovascular outcomes per 100 person-years, including recurrent events, are presented in Table 2. This finding demonstrates a significantly elevated rate of hospitalization for all CVD in patients with left-sided (1.72 per 100 person years) vs right-sided tumors (1.63 per 100 person years, *P* < .001), corresponding to a hazard ratio of 1.05 (95% CI, 1.00-1.11; *P* = .04). There was no difference in rates of hospitalization for AMI, stroke, HF, or pericardial disease. In the subgroup of women with preexisting CVD, there was also no difference in rates of hospitalization for any major CVD, AMI, stroke, or HF (eTable 9 in Supplement 1).

**Table 2.** Rates of Hospitalization for Cardiovascular Disease After Receiving Radiation Therapy for Left-Sided vs Right-Sided Breast Cancer

Hospitalization	Hospitalization rate per 100 person-years (95% CI)		*P* value
	Left	Right	
All major cardiovascular disease	1.72 (1.68-1.76)	1.63 (1.59-1.66)	<.001
Acute myocardial infarction	0.31 (0.29-0.33)	0.29 (0.27-0.31)	.08
Stroke	0.30 (0.28-0.31)	0.27 (0.26-0.29)	.05
Congestive heart failure	1.00 (0.97-1.03)	0.97 (0.94-1.00)	.17
Pericardial disease	0.08 (0.07-0.09)	0.07 (0.07-0.08)	.23
Revascularization with PCI	0.20 (0.18-0.21)	0.19 (0.18-0.20)	.52

Abbreviation: PCI, percutaneous coronary intervention.

## Discussion

In this population-based cohort study, left-sided BC was not associated with increased risk of first cardiovascular hospitalization or most cardiovascular outcomes. We observed similar patterns in the subgroup with prior CVD, who are expected to be at the highest risk based on the multiple-hit hypothesis.^26^ Among patients without prior CVD, women with left-sided tumors had a minimally increased risk of new HF and IHD. There was also an increased risk of pericardial disease limited to the first 0 to 2 years after radiotherapy. Stratified analyses suggest that radiation-associated CVD risk may be more relevant for women younger than 50 years and those who received chemotherapy. These findings provide population-level reassurance that modern radiation techniques have largely mitigated the excess long-term cardiovascular mortality and morbidity historically associated with left-sided BC treatment for women with typical radiation exposure.

The cardiovascular health of BC survivors has assumed increasing importance because they are at higher risk of CVD compared with cancer-free women.^27,28,29,30^ This makes it inappropriate to conduct comparisons of CVD risk after left-sided EBRT with cancer-free women. Instead, we focused our study on patients diagnosed with BC undergoing EBRT to allow for an assessment of the independent risk posed by left-sided EBRT. As we expected, tumor laterality was a random phenomenon that had no association with measurable baseline characteristics between women with left- or right-sided tumors.

### Prior Studies on Radiation Laterality and Mortality

In a large US cohort of women diagnosed with early-stage BC between 1973 and 2001, the excess cardiac mortality associated with left-sided radiation therapy decreased steadily across successive decades, although follow-up was limited for patients treated after 1992.^18^ A subsequent Japanese observational study of 12 911 women treated between 2000 and 2015 likewise found no significant difference in cardiac mortality between left- and right-sided disease (2.0% vs 1.7%, *P* = .43) during a median follow-up of 8.3 years.^31^ Several European and Chinese studies of patients treated before 2008 also showed no increase in cardiac mortality with contemporary left-sided EBRT.^19,20,21^ Our study builds on prior observations by studying women treated through 2017 in one of the most diverse populations in the world.^32^ We go beyond cardiovascular mortality (which is predisposed to misclassification and requires longer-term follow-up to detect differences) to demonstrate no significant difference in all-cause mortality when comparing women with left- vs right- sided BC receiving contemporary EBRT.

### Prior Studies on Radiation Laterality and CVD

Darby et al^5^ later reported a linear association between MHD and IHD in women treated from 1958 to 2001. A meta-analysis^33^ pooling results from 9 observational studies^34,35,36,37,38,39,40,41,42^ reported a 10% higher coronary risk for left- vs right-sided tumors, but this excess disappeared in the post-1980 subgroup. A study by van Velzen et al^43^ found that the mean heart dose for contemporary left-sided EBRT was less than 3 Gy. The authors also showed that in patients receiving less than 4 Gy (76% of the total cohort), there was no significant association between radiation dose and risk of IHD. This finding may explain the negligible increase in CVD risk with contemporary left-sided EBRT because the dose received by most women in recent years may not reach the threshold needed to provoke clinically overt CVD. In another retrospective cohort study^50^ of 972 women younger than 55 years diagnosed with BC between 1985 and 2008, left-sided radiation therapy was associated with an increased risk of CAD 15 years after radiation therapy. This finding aligns with our finding that having a left-sided tumor was associated with increased risk of hospitalization for any cardiovascular disease at 15 years after radiation treatment only in women younger than 50 years (and not those older than 50 years).

There are even fewer data pertaining to specific forms of CVD beyond CAD and IHD after left-sided EBRT. Studies from the US (1986-1993) and the Netherlands (1989-2004) found no significant differences in valvular disease, arrhythmias, or heart failure by laterality. Conversely, larger Scandinavian cohorts treated mainly before 2005 reported modestly higher risks of pericarditis, valvular disease, or overall CVD among women with left-sided tumors.^38,40^ Our study examines more recent data of women treated after 2002. The large sample size and long follow-up period of our study allowed us to detect statistical significance of small differences between groups, which may not be considered clinically meaningful, but it also increases the confidence in the absence of differences between groups for most CVD outcomes. We were also able to examine a wider variety of cardiovascular diagnoses along with cardiovascular mortality during longer follow-up than in most prior studies.

### Limitations

This study has some limitations. We cannot rule out the presence of confounding, despite the apparent random nature of tumor laterality and lack of differences in measured baseline characteristics between the compared groups. Our reliance on administrative data precluded access to some important patient and treatment characteristics, including radiation doses to the heart and treatment plans. This is relevant because some patients have unfavorable anatomy (heart closer to or within radiation field), resulting in higher-than-expected MHD, and the results from this cohort would not be applicable to them. There is, however, a movement away from the MHD model given that it is an imprecise marker of dose to cardiac substrates and predictor of cardiovascular outcomes.^44,45,46^ Additionally, we did not account for higher radiation doses associated with treatment of internal mammary nodes. Our study is less equipped to address the long-term effects of this practice, which likely increased after the 2015 MA.20 trial.^47^ We did not find significant differences in our primary outcome between patients with left- vs right-sided tumors when stratified by treatment era, including the 2013 to 2017 era, when internal mammary node radiation therapy became more widespread. One-third of our cohort had unknown stage (data available only for patients treated after 2007), but the proportion was balanced between patients with left- and right-sided tumors, so we do not anticipate this to meaningfully influence the association of tumor laterality with CVD. We were unable to differentiate between patients who received partial breast vs whole breast radiation therapy and were unable to study subclinical or imaging-based cardiac injury. Lastly, there are numerous outcomes presented within this article. Any analysis beyond the primary outcome should be interpreted as exploratory; this is particularly relevant for the stratified analyses, which were conducted post hoc after the peer review process.

## Conclusions

In this population-based cohort study, contemporary EBRT techniques were associated with small long-term increases in CVD risk for woman with left-sided BC, with no differences observed for most outcomes. These findings suggest that contemporary photon-based EBRT techniques have substantially reduced the cardiovascular risk historically associated with left-sided BC radiation therapy. These data may aid clinicians and patients when discussing the risks and benefits of EBRT, specifically in clinical settings where heart doses are expected to be low.

## Notes

Supplement 1. **eFigure.** Cumulative incidence function curves for pericardial disease and coronary artery revascularization outcomes after radiation therapy **eTable 1.** Baseline characteristics in patients with prior cardiovascular disease stratified by tumor laterality (N=4,911) **eTable 2.** Baseline characteristics in patients without prior cardiovascular disease stratified by tumor laterality (N=71,675) **eTable 3.** Summary of the results from a cause-specific hazards regression model evaluating the association of baseline characteristics with time to first hospitalization for cardiovascular disease **eTable 4.** Cumulative incidence (95% confidence interval) of pericardial disease at 0-2, 2-5, 5-10, and >10 years post-radiation intervals for left- versus right-sided breast cancer **eTable 5.** Summary of cumulative incidences for each outcome at 5-, 10- and 15-years post radiation by tumor laterality **eTable 6.** Cumulative incidence (95% confidence interval) for each outcome among women with prior cardiovascular disease at 15-years post-radiation for left- versus right-sided breast cancer **eTable 7.** Cumulative incidence (95% confidence interval) for hospitalization for all major cardiovascular disease at 5, 10, and 15-years post-radiation for left- versus right-sided breast cancer, stratified by age **eTable 8.** Cumulative incidence (95% confidence interval) for hospitalization for all major cardiovascular disease at 5, 10, and 15-years post-radiation for left- versus right-sided breast cancer, stratified by receipt of chemotherapy **eTable 9.** Rate of hospitalization (per 100 person years) (95% confidence interval) for cardiovascular disease in women with pre-existing cardiovascular disease after receiving radiation for left-sided versus right-sided breast cancer **eMethods.** Supplementary methods
eReferences

Supplement 2.
Data Sharing Statement

## References

[1] Darby S, McGale P, Correa C, ; Early Breast Cancer Trialists’ Collaborative Group (EBCTCG). Effect of radiotherapy after breast-conserving surgery on 10-year recurrence and 15-year breast cancer death: meta-analysis of individual patient data for 10,801 women in 17 randomised trials. Lancet. 2011;378(9804):1707-1716. doi:10.1016/S0140-6736(11)61629-2 22019144 PMC3254252

[2] McGale P, Taylor C, Correa C, ; EBCTCG (Early Breast Cancer Trialists’ Collaborative Group). Effect of radiotherapy after mastectomy and axillary surgery on 10-year recurrence and 20-year breast cancer mortality: meta-analysis of individual patient data for 8135 women in 22 randomised trials. Lancet. 2014;383(9935):2127-2135. doi:10.1016/S0140-6736(14)60488-8 24656685 PMC5015598

[3] Jagsi R, Pierce L. Postmastectomy radiation therapy for patients with locally advanced breast cancer. Semin Radiat Oncol. 2009;19(4):236-243. doi:10.1016/j.semradonc.2009.05.009 19732688

[4] Early Breast Cancer Trialists' Collaborative Group. Favourable and unfavourable effects on long-term survival of radiotherapy for early breast cancer: an overview of the randomised trials. Lancet. 2000;355(9217):1757-1770. doi:10.1016/S0140-6736(00)02263-7 10832826

[5] Darby SC, Ewertz M, McGale P, . Risk of ischemic heart disease in women after radiotherapy for breast cancer. N Engl J Med. 2013;368(11):987-998. doi:10.1056/NEJMoa1209825 23484825

[6] Paszat LF, Mackillop WJ, Groome PA, Boyd C, Schulze K, Holowaty E. Mortality from myocardial infarction after adjuvant radiotherapy for breast cancer in the surveillance, epidemiology, and end-results cancer registries. J Clin Oncol. 1998;16(8):2625-2631. doi:10.1200/JCO.1998.16.8.2625 9704712

[7] Rutqvist LE, Johansson H. Mortality by laterality of the primary tumour among 55,000 breast cancer patients from the Swedish Cancer Registry. Br J Cancer. 1990;61(6):866-868. doi:10.1038/bjc.1990.193 2372488 PMC1971705

[8] Belzile-Dugas E, Eisenberg MJ. Radiation-induced cardiovascular disease: review of an underrecognized pathology. J Am Heart Assoc. 2021;10(18):e021686. doi:10.1161/JAHA.121.021686 34482706 PMC8649542

[9] Smith BD, Pan IW, Shih YC, . Adoption of intensity-modulated radiation therapy for breast cancer in the United States. J Natl Cancer Inst. 2011;103(10):798-809. doi:10.1093/jnci/djr100 21525437

[10] Taylor CW, Nisbet A, McGale P, Darby SC. Cardiac exposures in breast cancer radiotherapy: 1950s-1990s. Int J Radiat Oncol Biol Phys. 2007;69(5):1484-1495. doi:10.1016/j.ijrobp.2007.05.034 18035211

[11] Campana F, Kirova YM, Rosenwald JC, . Breast radiotherapy in the lateral decubitus position: a technique to prevent lung and heart irradiation. Int J Radiat Oncol Biol Phys. 2005;61(5):1348-1354. doi:10.1016/j.ijrobp.2004.08.051 15817336

[12] Latty D, Stuart KE, Wang W, Ahern V. Review of deep inspiration breath-hold techniques for the treatment of breast cancer. J Med Radiat Sci. 2015;62(1):74-81. doi:10.1002/jmrs.96 26229670 PMC4364809

[13] Drost L, Yee C, Lam H, . A systematic review of heart dose in breast radiotherapy. Clin Breast Cancer. 2018;18(5):e819-e824. doi:10.1016/j.clbc.2018.05.010 29980429

[14] Taylor CW, Wang Z, Macaulay E, Jagsi R, Duane F, Darby SC. Exposure of the heart in breast cancer radiation therapy: a systematic review of heart doses published during 2003 to 2013. Int J Radiat Oncol Biol Phys. 2015;93(4):845-853. doi:10.1016/j.ijrobp.2015.07.2292 26530753

[15] MacDonald SM, Jimenez R, Paetzold P, . Proton radiotherapy for chest wall and regional lymphatic radiation; dose comparisons and treatment delivery. Radiat Oncol. 2013;8:71. doi:10.1186/1748-717X-8-71 23521809 PMC3627609

[16] Bekelman JE, Lu H, Pugh S, ; RadComp (Radiotherapy Comparative Effectiveness Consortium). Pragmatic randomised clinical trial of proton versus photon therapy for patients with non-metastatic breast cancer: the Radiotherapy Comparative Effectiveness (RadComp) Consortium trial protocol. BMJ Open. 2019;9(10):e025556. doi:10.1136/bmjopen-2018-025556 31619413 PMC6797426

[17] Kirby AM, Haviland JS, Mackenzie M, ; PARABLE Trial Management Group. Proton beam therapy in breast cancer patients: the UK PARABLE trial is recruiting. Clin Oncol (R Coll Radiol). 2023;35(6):347-350. doi:10.1016/j.clon.2023.02.015 36933970

[18] Darby SC, McGale P, Taylor CW, Peto R. Long-term mortality from heart disease and lung cancer after radiotherapy for early breast cancer: prospective cohort study of about 300,000 women in US SEER cancer registries. Lancet Oncol. 2005;6(8):557-565. doi:10.1016/S1470-2045(05)70251-5 16054566

[19] Li WH, Zhang ZG, Huang ZR, Zhang W, Li ZB, Qi ZQ. No association between tumor laterality and cardiac-related mortality in breast cancer patients after radiotherapy: a population-based study. Cancer Manag Res. 2018;10:3649-3656. doi:10.2147/CMAR.S172595 30271213 PMC6152604

[20] Merzenich H, Baaken D, Schmidt M, . Cardiac late effects after modern 3D-conformal radiotherapy in breast cancer patients: a retrospective cohort study in Germany (ESCaRa). Breast Cancer Res Treat. 2022;191(1):147-157. doi:10.1007/s10549-021-06412-3 34626275 PMC8758608

[21] Rutter CE, Chagpar AB, Evans SB. Breast cancer laterality does not influence survival in a large modern cohort: implications for radiation-related cardiac mortality. Int J Radiat Oncol Biol Phys. 2014;90(2):329-334. doi:10.1016/j.ijrobp.2014.06.030 25304793

[22] Robles SC, Marrett LD, Clarke EA, Risch HA. An application of capture-recapture methods to the estimation of completeness of cancer registration. J Clin Epidemiol. 1988;41(5):495-501. doi:10.1016/0895-4356(88)90052-2 3367181

[23] Schull MJ, Azimaee M, Marra M, . ICES: data, discovery, better health. Int J Popul Data Sci. 2020;4(2):1135. 32935037 10.23889/ijpds.v4i2.1135 PMC7477779

[24] Personal Health Information Protection Act, 2004, S.O. 2004, c. 3, Sched. A. 2014.

[25] Austin PC. Using the standardized difference to compare the prevalence of a binary variable between two groups in observational research. Commun Stat Simul Comput. 2009;38(6):1228-1234. doi:10.1080/03610910902859574

[26] Jones LW, Haykowsky MJ, Swartz JJ, Douglas PS, Mackey JR. Early breast cancer therapy and cardiovascular injury. J Am Coll Cardiol. 2007;50(15):1435-1441. doi:10.1016/j.jacc.2007.06.037 17919562

[27] Abdel-Qadir H, Amir E, Fischer HD, . The risk of myocardial infarction with aromatase inhibitors relative to tamoxifen in post-menopausal women with early stage breast cancer. Eur J Cancer. 2016;68:11-21. doi:10.1016/j.ejca.2016.08.022 27693889

[28] Abdel-Qadir H, Ethier JL, Lee DS, Thavendiranathan P, Amir E. Cardiovascular toxicity of angiogenesis inhibitors in treatment of malignancy: A systematic review and meta-analysis. Cancer Treat Rev. 2017;53:120-127. doi:10.1016/j.ctrv.2016.12.002 28104567

[29] Abdel-Qadir H, Thavendiranathan P, Austin PC, . The risk of heart failure and other cardiovascular hospitalizations after early stage breast cancer: a matched cohort study. J Natl Cancer Inst. 2019;111(8):854-862. doi:10.1093/jnci/djy218 30715404 PMC6695318

[30] Abdel-Qadir H, Thavendiranathan P, Fung K, . Association of early-stage breast cancer and subsequent chemotherapy with risk of atrial fibrillation. JAMA Netw Open. 2019;2(9):e1911838. doi:10.1001/jamanetworkopen.2019.11838 31539076 PMC6755537

[31] Jingu K, Umezawa R, Yamamoto T, . Recent postoperative radiotherapy for left-sided breast cancer does not increase mortality of heart disease in Asians or Pacific Islanders: SEER database analysis. Anticancer Res. 2023;43(8):3571-3577. doi:10.21873/anticanres.16535 37500140

[32] Statistics Canada. Census profile, 2021 census of population — Ontario [province]. Government of Canada. Accessed February 18, 2026. https://www12.statcan.gc.ca/census-recensement/2021/dp-pd/prof/details/page.cfm?DGUIDlist=2021A000235&GENDERlist=1%2C2%2C3&HEADERlist=0&Lang=E&STATISTIClist=1

[33] Seth L, Makram O, Essa A, . Laterality of radiation therapy in breast cancer is not associated with increased risk of coronary artery disease in the contemporary era. Adv Radiat Oncol. 2024;9(10):101583. doi:10.1016/j.adro.2024.101583 39258143 PMC11385753

[34] Boekel NB, Schaapveld M, Gietema JA, . Cardiovascular morbidity and mortality after treatment for ductal carcinoma in situ of the breast. J Natl Cancer Inst. 2014;106(8):dju156. doi:10.1093/jnci/dju156 25128694 PMC4151854

[35] Borger JH, Hooning MJ, Boersma LJ, . Cardiotoxic effects of tangential breast irradiation in early breast cancer patients: the role of irradiated heart volume. Int J Radiat Oncol Biol Phys. 2007;69(4):1131-1138. doi:10.1016/j.ijrobp.2007.04.042 17606332

[36] Højris I, Overgaard M, Christensen JJ, Overgaard J; Radiotherapy Committee of the Danish Breast Cancer Cooperative Group. Morbidity and mortality of ischaemic heart disease in high-risk breast-cancer patients after adjuvant postmastectomy systemic treatment with or without radiotherapy: analysis of DBCG 82b and 82c randomised trials. Lancet. 1999;354(9188):1425-1430. doi:10.1016/S0140-6736(99)02245-X 10543669

[37] Hooning MJ, Botma A, Aleman BM, . Long-term risk of cardiovascular disease in 10-year survivors of breast cancer. J Natl Cancer Inst. 2007;99(5):365-375. doi:10.1093/jnci/djk064 17341728

[38] McGale P, Darby SC, Hall P, . Incidence of heart disease in 35,000 women treated with radiotherapy for breast cancer in Denmark and Sweden. Radiother Oncol. 2011;100(2):167-175. doi:10.1016/j.radonc.2011.06.016 21752480

[39] Patt DA, Goodwin JS, Kuo YF, . Cardiac morbidity of adjuvant radiotherapy for breast cancer. J Clin Oncol. 2005;23(30):7475-7482. doi:10.1200/JCO.2005.13.755 16157933

[40] Rehammar JC, Jensen MB, McGale P, . Risk of heart disease in relation to radiotherapy and chemotherapy with anthracyclines among 19,464 breast cancer patients in Denmark, 1977-2005. Radiother Oncol. 2017;123(2):299-305. doi:10.1016/j.radonc.2017.03.012 28365142 PMC5446317

[41] Wadsten C, Wennstig AK, Garmo H, . Risk of ischemic heart disease after radiotherapy for ductal carcinoma in situ. Breast Cancer Res Treat. 2018;171(1):95-101. doi:10.1007/s10549-018-4803-1 29730730

[42] Wennstig AK, Wadsten C, Garmo H, . Long-term risk of ischemic heart disease after adjuvant radiotherapy in breast cancer: results from a large population-based cohort. Breast Cancer Res. 2020;22(1):10. doi:10.1186/s13058-020-1249-2 31969169 PMC6977272

[43] van Velzen SGM, Gal R, Teske AJ, . AI-based radiation dose quantification for estimation of heart disease risk in breast cancer survivors after radiation therapy. Int J Radiat Oncol Biol Phys. 2022;112(3):621-632. doi:10.1016/j.ijrobp.2021.09.008 34624460

[44] Bowen Jones S, Marchant T, Saunderson C, McWilliam A, Banfill K. Moving beyond mean heart dose: The importance of cardiac substructures in radiation therapy toxicity. J Med Imaging Radiat Oncol. 2024;68(8):974-986. doi:10.1111/1754-9485.13737 39228181 PMC11686456

[45] Walls GM, Bergom C, Mitchell JD, Cardiotoxicity following thoracic radiotherapy for lung cancer. Br J Cancer. 2025;132(4):311-325. 10.1038/s41416-024-02888-0 PMC11833127 39506136

[46] Zhang SC, Nikolova AP, Kamrava M, Mak RH, Atkins KM. A roadmap for modelling radiation-induced cardiac disease. J Med Imaging Radiat Oncol. 2024;68(8):950-961. doi:10.1111/1754-9485.13716 38985978

[47] Whelan TJ, Olivotto IA, Parulekar WR, . Regional nodal irradiation in early-stage breast cancer. N Engl J Med. 2015;373(4):307-316. doi:10.1056/NEJMoa1415340 PMC4556358 26200977

[48] Merzenich H, Baaken D, Schmidt M, . Cardiac late effects after modern 3D-conformal radiotherapy in breast cancer patients: a retrospective cohort study in Germany (ESCaRa). Breast Cancer Res Treat. 2022;191(1):147-157. doi:10.1007/s10549-021-06412-3 34626275 PMC8758608

[49] Li WH, Zhang ZG, Huang ZR, Zhang W, Li ZB, Qi ZQ. No association between tumor laterality and cardiac-related mortality in breast cancer patients after radiotherapy: a population-based study. Cancer Manag Res. 2018;10:3649-3656. doi:10.2147/CMAR.S172595 30271213 PMC6152604

[50] Carlson LE, Watt GP, Tonorezos ES, . Coronary artery disease in young women after radiation therapy for breast cancer: the WECARE study. JACC Cardio Oncol. 2021;3(3):381-392. doi:10.1016/j.jaccao.2021.07.008 PMC8463731 34604798

