# Errors in Abstract and Table 1

**DOI:** 10.1001/jamanetworkopen.2026.17820

**Published:** 2026-05-15

In the Original Investigation titled “Long-Term Risk of Cardiovascular Disease After Contemporary Left-Sided Breast Radiation Therapy,”^1^ published April 1, 2026, there were errors in the Abstract and Table 1. The results section of the Abstract stated, “In women with preexisting CVD, new diagnoses of heart failure (10.2% [95% CI, 9.9%-10.6%] vs 9.6% [95% CI, 9.2%-10.0%]; *P*  = .01) and ischemic heart disease (13.6% [95% CI, 13.2%-14.0%] vs 12.8% [95% CI, 12.4%-13.2%]; *P*  = .03) were slightly more frequent after left-sided EBRT.” The first part of this statement should instead read as “In women without preexisting CVD,” to accurately reflect the subgroup analysis in women without cardiovascular disease at baseline. Additionally in the results section of the Abstract, in the statement that “The rate of CVD hospitalizations when including recurrent events was modestly higher for left-sided disease (1.72 vs 1.63 per 100 person-years; *P* = .006)”, the *P* value should instead read as *P* < .001. In Table 1, the No. (%) of patients listed in the Total column for acute myocardial infarction, hospitalization for stroke, atrial fibrillation, and valvular disease should have been 551 (0.7), 310 (0.4), 2991 (3.9), and 370 (0.5), respectively. This article has been corrected.^1^

## References

[1] Nakajima E, Nguyen L, Liu N, . Long-term risk of cardiovascular disease after contemporary left-sided breast radiation therapy. JAMA Netw Open. 2026;9(4):e264098. doi:10.1001/jamanetworkopen.2026.4098 41920543 PMC13044663

