# Supplementary material for: Long-Term Risk of Cardiovascular Disease After Contemporary Left-Sided Breast Radiation Therapy
Source: JAMA Netw Open. 2026 Apr 1;9(4):e264098. doi: 10.1001/jamanetworkopen.2026.4098 (PMC13044663; doi:10.1001/jamanetworkopen.2026.4098)
Supplement: Supplement 1. — eFigure. Cumulative incidence function curves for pericardial disease and coronary artery revascularization outcomes after radiation therapy eTable 1. Baseline characteristics in patients with prior cardiovascular disease stratified by tumor laterality (N=4,911) eTable 2. Baseline characteristics in patients without prior cardiovascular disease stratified by tumor laterality (N=71,675) eTable 3. Summary of the results from a cause-specific hazards regression model evaluating the association of baseline characteristics with time to first hospitalization for cardiovascular disease eTable 4. Cumulative incidence (95% confidence interval) of pericardial disease at 0-2, 2-5, 5-10, and >10 years post-radiation intervals for left- versus right-sided breast cancer eTable 5. Summary of cumulative incidences for each outcome at 5-, 10- and 15-years post radiation by tumor laterality eTable 6. Cumulative incidence (95% confidence interval) for each outcome among women with prior cardiovascular disease at 15-years post-radiation for left- versus right-sided breast cancer eTable 7. Cumulative incidence (95% confidence interval) for hospitalization for all major cardiovascular disease at 5, 10, and 15-years post-radiation for left- versus right-sided breast cancer, stratified by age eTable 8. Cumulative incidence (95% confidence interval) for hospitalization for all major cardiovascular disease at 5, 10, and 15-years post-radiation for left- versus right-sided breast cancer, stratified by receipt of chemotherapy eTable 9. Rate of hospitalization (per 100 person years) (95% confidence interval) for cardiovascular disease in women with pre-existing cardiovascular disease after receiving radiation for left-sided versus right-sided breast cancer eMethods. Supplementary methods eReferences [file jamanetwopen-e264098-s001.pdf]

Supplemental Online Content

Nakajima E, Nguyen L, Liu N, et al. Long-term risk of cardiovascular disease after contemporary left-sided breast radiation therapy. *JAMA Netw Open*. 2026;9(4):e264098. doi:10.1001/jamanetworkopen.2026.4098

eFigure. Cumulative incidence function curves for pericardial disease and coronary artery revascularization outcomes after radiation therapy
eTable 1. Baseline characteristics in patients with prior cardiovascular disease stratified by tumor laterality (N=4,911)
eTable 2. Baseline characteristics in patients without prior cardiovascular disease stratified by tumor laterality (N=71,675)
eTable 3. Summary of the results from a cause-specific hazards regression model evaluating the association of baseline characteristics with time to first hospitalization for cardiovascular disease
eTable 4. Cumulative incidence (95% confidence interval) of pericardial disease at 0-2, 2-5, 5-10, and >10 years post-radiation intervals for left- versus right-sided breast cancer
eTable 5. Summary of cumulative incidences for each outcome at 5-, 10- and 15-years post radiation by tumor laterality
eTable 6. Cumulative incidence (95% confidence interval) for each outcome among women with prior cardiovascular disease at 15-years post-radiation for left- versus right-sided breast cancer
eTable 7. Cumulative incidence (95% confidence interval) for hospitalization for all major cardiovascular disease at 5, 10, and 15-years post-radiation for left- versus right-sided breast cancer, stratified by age
eTable 8. Cumulative incidence (95% confidence interval) for hospitalization for all major cardiovascular disease at 5, 10, and 15-years post-radiation for left- versus right-sided breast cancer, stratified by receipt of chemotherapy
eTable 9. Rate of hospitalization (per 100 person years) (95% confidence interval) for cardiovascular disease in women with pre-existing cardiovascular disease after receiving radiation for left-sided versus right-sided breast cancer
eMethods. Supplementary methods
eReferences

This supplemental material has been provided by the authors to give readers additional information about their work.

© 2026 Nakajima E et al. *JAMA Network Open*.

eFigure. Cumulative incidence function curves for pericardial disease and coronary artery revascularization outcomes after radiation therapy

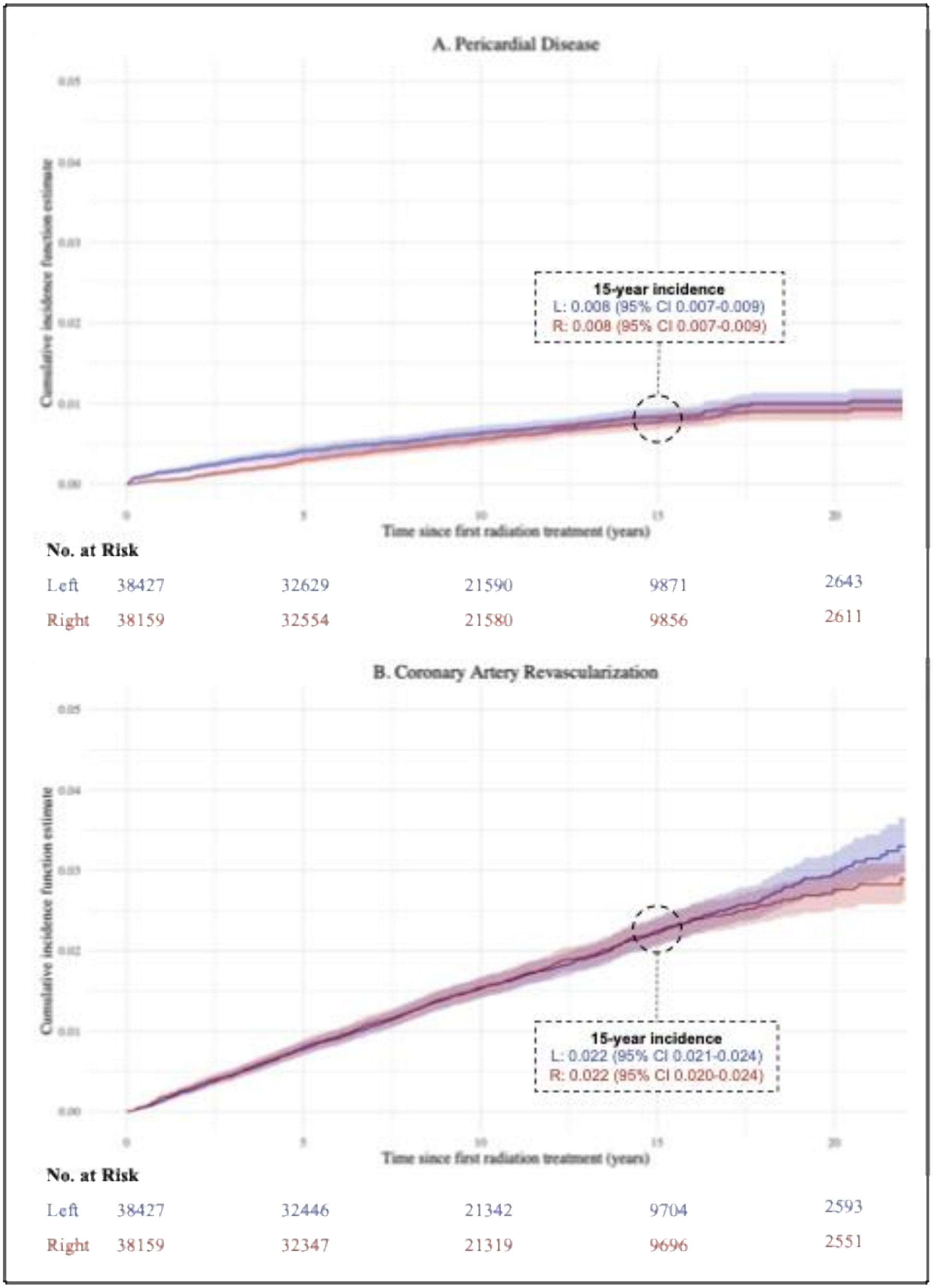

© 2026 Nakajima E et al. *JAMA Network Open*.

eTable 1. Baseline characteristics in patients with prior cardiovascular disease stratified by tumor laterality (N=4,911)

	Total (N=4,911)	Left (N=2,448)	Right (N=2,463)	St Diff	P value
**Mean age at first RT (y, SD)**	69.5 (11.2)	69.3 (11.3)	69.6 (11.1)	0.03	0.32
**Median follow-up time (y, IQR)**	8.5 (4.4-12.2)	8.4 (4.0-12.2)	8.6 (4.8-12.1)	0.04	0.19
**Reason for Censoring (n, %)**					
Death	2,285 (46.5)	1,122 (45.8)	1,163 (47.2)	0.03	0.41
Second Cancer Diagnosis	671 (13.7)	349 (14.3)	322 (13.1)	0.03	
End of study period	1,955 (39.8)	977 (39.9)	978 (39.7)	<0.01	
**Year of first RT (n, %)**					0.10
2002	251 (5.1)	114 (4.7)	137 (5.6)	0.04	
2003	244 (5.0)	141 (5.8)	103 (4.2)	0.07	
2004	242 (4.9)	130 (5.3)	112 (4.5)	0.04	
2005	262 (5.3)	144 (5.9)	118 (4.8)	0.05	
2006	258 (5.3)	121 (4.9)	137 (5.6)	0.03	
2007	258 (5.3)	134 (5.5)	124 (5.0)	0.02	
2008	273 (5.6)	137 (5.6)	136 (5.5)	<0.01	
2009	280 (5.7)	134 (5.5)	146 (5.9)	0.02	
2010	308 (6.3)	147 (6.0)	161 (6.5)	0.02	
2011	350 (7.1)	171 (7.0)	179 (7.3)	0.01	
2012	363 (7.4)	179 (7.3)	184 (7.5)	0.01	
2013	381 (7.8)	176 (7.2)	205 (8.3)	0.04	
2014	399 (8.1)	181 (7.4)	218 (8.9)	0.05	
2015	359 (7.3)	186 (7.6)	173 (7.0)	0.02	
2016	381 (7.8)	202 (8.3)	179 (7.3)	0.04	
2017	302 (6.1)	151 (6.2)	151 (6.1)	<0.01	
**Neighborhood Income Quintile (n, %)**					0.39
I	950 (19.3)	490 (20.0)	460 (18.7)	0.03	
II	1,034 (21.1)	493 (20.1)	541 (22.0)	0.05	
III	902 (18.4)	468 (19.1)	434 (17.6)	0.04	
IV	1,017 (20.7)	499 (20.4)	518 (21.0)	0.02	
V	992 (20.2)	489 (20.0)	503 (20.4)	0.01	
**Rural Residence (n, %)**	387 (7.9)	182 (7.4)	205 (8.3)	0.03	0.32
**Neighborhood Racialized and Newcomer Populations Quintile (n, %)**					0.24
I	1,001 (20.4)	484 (19.8)	517 (21.0)	0.03	
II	1,048 (21.3)	499 (20.4)	549 (22.3)	0.05	
III	967 (19.7)	480 (19.6)	487 (19.8)	<0.01	
IV	960 (19.5)	497 (20.3)	463 (18.8)	0.04	
V	879 (17.9)	457 (18.7)	422 (17.1)	0.04	
**Stage (n, %)**					0.48
I	1,545 (31.5)	748 (30.6)	797 (32.4)	0.04	
II	1,364 (27.8)	678 (27.7)	686 (27.9)	<0.01	

© 2026 Nakajima E et al. *JAMA Network Open.*

III	647 (13.2)	329 (13.4)	318 (12.9)	0.02	
Unknown	1,355 (27.6)	693 (28.3)	662 (26.9)	0.03	
**Surgery type (n, %)**					0.44
Breast Conserving Surgery	3,594 (73.2)	1,771 (72.3)	1,823 (74.0)	0.04	
Partial Mastectomy	11 (0.2)	*6-10	*1-5	0.03	
Mastectomy	939 (19.1)	486 (19.9)	453 (18.4)	0.04	
None	367 (7.5)	*181-185	*182-186	<0.01	
**Chemotherapy (n, %)**	1,937 (39.4)	988 (40.4)	949 (38.5)	0.04	0.19
**Trastuzumab (n, %)**	364 (7.4)	182 (7.4)	182 (7.4)	<0.01	0.95
**Mean time from diagnosis to RT (days, SD)**	156.2 (94.9)	157.6 (95.6)	154.7 (94.3)	0.03	0.29
**Diabetes (n, %)**	1,478 (30.1)	733 (29.9)	745 (30.2)	0.01	0.82
**Hypertension (n, %)**	3,811 (77.6)	1,887 (77.1)	1,924 (78.1)	0.03	0.39
**Chronic Obstructive Pulmonary Disorder (n, %)**	588 (12.0)	302 (12.3)	286 (11.6)	0.02	0.43
**Chronic Kidney Disease (n, %)**	425 (8.7)	235 (9.6)	190 (7.7)	0.07	0.02
**Acute Myocardial Infarction (n, %)**	551 (11.2)	279 (11.4)	272 (11.0)	0.01	0.69
**Stroke hospitalization (n, %)**	310 (6.3)	147 (6.0)	163 (6.6)	0.03	0.38
**Heart Failure (n, %)**	1,930 (39.3)	971 (39.7)	959 (38.9)	0.02	0.60
**Ischemic Heart Disease (n, %)**	2,540 (51.7)	1,279 (52.2)	1,261 (51.2)	0.02	0.46
**Atrial Fibrillation (n, %)**	2,991 (60.9)	1,468 (60.0)	1,523 (61.8)	0.04	0.18
**Valvular disease (n, %)**	370 (7.5)	200 (8.2)	170 (6.9)	0.05	0.09

RT: radiation therapy, SD: standard deviation, IQR: interquartile range
*Denotes small cells

eTable 2. Baseline characteristics in patients without prior cardiovascular disease stratified by tumor laterality (N=71,675)

	Total (N=71,675)	Left (N=35,979)	Right (N=35,696)	St Diff	P value
**Mean age at first RT (y, SD)**	58.5 (11.9)	58.6 (12.0)	58.4 (11.9)	0.01	0.10
**Median follow-up time (y, IQR)**	11.1 (7.8-15.4)	11.1 (7.8-15.4)	11.1 (7.8-15.4)	<0.01	0.45
**Reason for Censoring (n, %)**					0.31
Death	15,369 (21.4)	7,795 (21.7)	7,574 (21.2)	0.01	
Second Cancer Diagnosis	10,400 (14.5)	5,184 (14.4)	5,216 (14.6)	<0.01	
End of study period	45,906 (64.0)	23,000 (63.9)	22,906 (64.2)	<0.01	
**Year of first RT (n, %)**					0.65
2002	3,964 (5.5)	1,996 (5.5)	1,968 (5.5)	<0.01	
2003	3,702 (5.2)	1,860 (5.2)	1,842 (5.2)	<0.01	
2004	3,754 (5.2)	1,926 (5.4)	1,828 (5.1)	0.01	
2005	4,184 (5.8)	2,076 (5.8)	2,108 (5.9)	0.01	
2006	4,280 (6.0)	2,095 (5.8)	2,185 (6.1)	0.01	
2007	4,152 (5.8)	2,122 (5.9)	2,030 (5.7)	0.01	
2008	4,306 (6.0)	2,191 (6.1)	2,115 (5.9)	0.01	
2009	4,441 (6.2)	2,191 (6.1)	2,250 (6.3)	0.01	
2010	4,613 (6.4)	2,341 (6.5)	2,272 (6.4)	0.01	
2011	4,851 (6.8)	2,440 (6.8)	2,411 (6.8)	<0.01	
2012	4,915 (6.9)	2,445 (6.8)	2,470 (6.9)	0.01	
2013	4,918 (6.9)	2,481 (6.9)	2,437 (6.8)	<0.01	
2014	5,131 (7.2)	2,540 (7.1)	2,591 (7.3)	0.01	
2015	5,125 (7.2)	2,582 (7.2)	2,543 (7.1)	<0.01	
2016	5,333 (7.4)	2,710 (7.5)	2,623 (7.3)	0.01	
2017	4,006 (5.6)	1,983 (5.5)	2,023 (5.7)	0.01	
**Neighborhood Income Quintile (n, %)**					0.17
I	11,805 (16.5)	6,008 (16.7)	5,797 (16.2)	0.01	
II	13,609 (19.0)	6,851 (19.0)	6,758 (18.9)	<0.01	
III	14,076 (19.6)	6,979 (19.4)	7,097 (19.9)	0.01	
IV	15,152 (21.1)	7,679 (21.3)	7,473 (20.9)	0.01	
V	16,880 (23.6)	8,386 (23.3)	8,494 (23.8)	0.01	
**Rural Residence (n, %)**	5,448 (7.6)	2,672 (7.4)	2,776 (7.8)	0.01	0.20
**Neighborhood Racialized and Newcomer Populations Quintile (n, %)**					0.61
I	13,154 (18.4)	6,599 (18.3)	6,555 (18.4)	<0.01	
II	13,566 (18.9)	6,761 (18.8)	6,805 (19.1)	0.01	
III	14,169 (19.8)	7,054 (19.6)	7,115 (19.9)	0.01	
IV	14,541 (20.3)	7,367 (20.5)	7,174 (20.1)	0.01	
V	15,647 (21.8)	7,891 (21.9)	7,756 (21.7)	0.01	
**Stage (n, %)**					0.39
I	22,468 (31.3)	11,232 (31.2)	11,236 (31.5)	0.01	
II	19,437 (27.1)	9,853 (27.4)	9,584 (26.8)	0.01	

© 2026 Nakajima E et al. *JAMA Network Open.*

III	8,134 (11.3)	4,094 (11.4)	4,040 (11.3)	<0.01	
Unknown	21,636 (30.2)	10,800 (30.0)	10,836 (30.4)	0.01	
**Surgery type (n, %)**					0.38
Breast Conserving Surgery	53,966 (75.3)	27,114 (75.4)	26,852 (75.2)	<0.01	
Partial Mastectomy	222 (0.3)	113 (0.3)	109 (0.3)	<0.01	
Mastectomy	12,884 (18.0)	6,497 (18.1)	6,387 (17.9)	<0.01	
None	4,603 (6.4)	2,255 (6.3)	2,348 (6.6)	0.01	
**Chemotherapy (n, %)**	40,372 (56.3)	20,409 (56.7)	19,963 (55.9)	0.02	0.03
**Trastuzumab (n, %)**	6,715 (9.4)	3,455 (9.6)	3,260 (9.1)	0.02	0.03
**Mean time from diagnosis to RT (days, SD)**	166.5 (83.2)	167.2 (83.3)	165.9 (83.1)	0.02	0.03
**Diabetes (n, %)**	8,970 (12.5)	4,553 (12.7)	4,417 (12.4)	0.01	0.26
**Hypertension (n, %)**	28,789 (40.2)	14,550 (40.4)	14,239 (39.9)	0.01	0.13
**Chronic Obstructive Pulmonary Disorder (n, %)**	2,199 (3.1)	1,079 (3.0)	1,120 (3.1)	0.01	0.28
**Chronic Kidney Disease (n, %)**	1,040 (1.5)	499 (1.4)	541 (1.5)	0.01	0.15

RT: radiation therapy, SD: standard deviation, IQR: interquartile range

eTable 3. Summary of the results from a cause-specific hazards regression model evaluating the association of baseline characteristics with time to first hospitalization for cardiovascular disease

Covariate	Hazard Ratio (95 CI)	P-value
Tumor Laterality (Left vs. Right)	1.02 (0.98, 1.06)	0.36
Age (per 1 year increase)	1.05 (1.05, 1.05)	<0.001
Year of RT (per 1 year increase)	0.96 (0.96, 0.97)	<0.001
History of Diabetes	1.47 (1.40, 1.55)	<0.001
History of Hypertension	1.58 (1.51, 1.66)	<0.001
Surgery Type		
Mastectomy (vs. BCS)	0.98 (0.93, 1.04)	0.52
No surgery (vs. BCS)	0.87 (0.79, 0.96)	<0.001
Partial Mastectomy (vs. BCS)	0.87 (0.62, 1.23)	0.44
Received Chemotherapy	1.11 (1.06, 1.16)	<0.001

CI: confidence interval, RT: radiation therapy, BCS: breast conserving surgery

eTable 4. Cumulative incidence (95% confidence interval) of pericardial disease at 0-2, 2-5, 5-10, and >10 years post-radiation intervals for left- versus right-sided breast cancer

	Left	Right	P-value
0-2 Years	0.002 (0.002, 0.003)	0.001 (0.001, 0.001)	<0.001
2-5 years	0.002 (0.002, 0.003)	0.002 (0.002, 0.003)	0.80
5-10 years	0.003 (0.002, 0.003)	0.003 (0.002, 0.003)	0.46
>10 years	0.005 (0.004, 0.006)	0.005 (0.003, 0.006)	0.91

© 2026 Nakajima E et al. *JAMA Network Open.*

eTable 5. Summary of cumulative incidences for each outcome at 5-, 10- and 15-years post radiation by tumor laterality.

	Left-sided BC	Right-sided BC	P value
Hospitalization for all major CVD			
5 years	0.050 (0.048-0.052)	0.048 (0.046-0.050)	0.18
10 years	0.094 (0.091-0.097)	0.093 (0.090-0.096)	0.60
15 years	0.138 (0.134-0.142)	0.135 (0.131-0.139)	0.43
All-cause mortality			
5 years	0.103 (0.100-0.106)	0.099 (0.096-0.102)	0.06
10 years	0.180 (0.176-0.184)	0.177 (0.173-0.182)	0.21
15 years	0.261 (0.255-0.266)	0.260 (0.255-0.265)	0.43
Hospitalization for AMI			
5 years	0.011 (0.010-0.012)	0.011 (0.010-0.012)	0.62
10 years	0.022 (0.020-0.023)	0.022 (0.020-0.023)	0.99
15 years	0.032 (0.030-0.035)	0.032 (0.030-0.035)	0.93
Hospitalization for stroke			
5 years	0.008 (0.008-0.009)	0.008 (0.007-0.009)	0.29
10 years	0.017 (0.015-0.018)	0.016 (0.015-0.017)	0.41
15 years	0.028 (0.026-0.030)	0.026 (0.024-0.028)	0.14
Hospitalization for pericardial disease			
5 years	0.004 (0.003-0.005)	0.003 (0.003-0.004)	0.01
10 years	0.006 (0.006-0.007)	0.006 (0.005-0.006)	0.11
15 years	0.008 (0.007-0.009)	0.008 (0.007-0.009)	0.20
Revascularization of CAD with PCI			
5 years	0.008 (0.007-0.009)	0.008 (0.007-0.009)	0.68
10 years	0.015 (0.014-0.017)	0.015 (0.014-0.017)	0.86
15 years	0.022 (0.021-0.024)	0.022 (0.020-0.024)	0.97
New Diagnosis of CHF			
5 years	0.030 (0.029-0.032)	0.028 (0.026-0.030)	0.04
10 years	0.062 (0.060-0.065)	0.058 (0.056-0.061)	0.02
15 years	0.102 (0.099-0.106)	0.096 (0.092-0.100)	0.01
New Diagnosis of IHD			
5 years	0.056 (0.054-0.059)	0.057 (0.054-0.059)	0.77
10 years	0.098 (0.094-0.101)	0.094 (0.091-0.097)	0.13
15 years	0.136 (0.132-0.140)	0.128 (0.124-0.132)	0.03
New Diagnosis of AF			
5 years	0.027 (0.026-0.029)	0.027 (0.025-0.028)	0.60
10 years	0.062 (0.059-0.064)	0.059 (0.057-0.062)	0.20
15 years	0.043 (0.040-0.046)	0.043 (0.040-0.045)	0.71
MACE Composite			
5 years	0.026 (0.024-0.027)	0.025 (0.024-0.027)	0.75
10 years	0.052 (0.050-0.055)	0.053 (0.051-0.055)	0.84
15 years	0.084 (0.081-0.088)	0.083 (0.080-0.087)	0.95
Cardiac-Specific Composite			
5 years	0.051 (0.049-0.054)	0.051 (0.048-0.053)	0.66

© 2026 Nakajima E et al. *JAMA Network Open.*

10 years	0.095 (0.092-0.098)	0.093 (0.091-0.096)	0.63
15 years	0.136 (0.132-0.140)	0.133 (0.129-0.137)	0.44

CVD: cardiovascular disease, AMI: acute myocardial infarction, CAD: coronary artery disease, PCI: percutaneous coronary intervention, CHF: congestive heart failure, IHD: ischemic heart disease, AF: atrial fibrillation, MACE: major adverse cardiovascular event

eTable 6. Cumulative incidence (95% confidence interval) for each outcome among women with prior cardiovascular disease at 15-years post-radiation for left- versus right-sided breast cancer

	Left	Right	P-value
**All-Cause Mortality**	0.548 (0.522-0.574)	0.573 (0.546-0.598)	0.86
**Hospitalization**			
All Major Cardiovascular Disease	0.352 (0.333-0.375)	0.358 (0.336-0.380)	0.86
Acute Myocardial Infarction	0.079 (0.067-0.092)	0.082 (0.070-0.096)	0.80
Stroke	0.059 (0.049-0.072)	0.061 (0.050-0.073)	0.44
Congestive Heart Failure	0.259 (0.239-0.280)	0.290 (0.268-0.312)	0.14

eTable 7. Cumulative incidence (95% confidence interval) for hospitalization for all major cardiovascular disease at 5, 10, and 15-years post-radiation for left- versus right-sided breast cancer, stratified by age

	Left	Right	P-value
**Age < 50 years**			
5 years	0.019 (0.017, 0.023)	0.015 (0.013, 0.018)	0.04
10 years	0.032 (0.028, 0.036)	0.028 (0.024, 0.032)	0.12
15 years	0.048 (0.043, 0.053)	0.039 (0.034, 0.043)	0.02
**Age 50-64 years**			
5 years	0.032 (0.030, 0.035)	0.031 (0.028, 0.034)	0.44
10 years	0.065 (0.061, 0.069)	0.064 (0.060, 0.068)	0.53
15 years	0.105 (0.099, 0.111)	0.103 (0.097, 0.109)	0.64
**Age > 64 years**			
5 years	0.093 (0.088, 0.098)	0.092 (0.087, 0.097)	0.90
10 years	0.173 (0.167, 0.180)	0.178 (0.171, 0.185)	0.49
15 years	0.248 (0.239, 0.257)	0.251 (0.242, 0.260)	0.60

eTable 8. Cumulative incidence (95% confidence interval) for hospitalization for all major cardiovascular disease at 5, 10, and 15-years post-radiation for left- versus right-sided breast cancer, stratified by receipt of chemotherapy

	Left	Right	P-value
**No chemotherapy**			
5 years	0.062 (0.059, 0.066)	0.062 (0.058, 0.066)	0.90
10 years	0.118 (0.113, 0.123)	0.121 (0.116, 0.126)	0.41
15 years	0.168 (0.162, 0.175)	0.174 (0.168, 0.181)	0.28
**Received chemotherapy**			
5 years	0.040 (0.038, 0.043)	0.036 (0.034, 0.039)	0.04
10 years	0.075 (0.072, 0.079)	0.070 (0.067, 0.074)	0.04
15 years	0.113 (0.108, 0.119)	0.103 (0.098, 0.108)	0.01

eTable 9. Rate of hospitalization (per 100 person years) (95% confidence interval)for cardiovascular disease in women with pre-existing cardiovascular disease after receiving radiation for left-sided versus right-sided breast cancer

	Left	Right	P-value
**Hospitalization**			
All Major Cardiovascular Disease	6.97 (6.62-7.33)	6.71 (6.38-7.07)	0.31
Acute Myocardial Infarction	0.97 (0.85-1.11)	0.96 (0.84-1.10)	0.93
Stroke	0.84 (0.73-0.97)	0.98 (0.86-1.13)	0.12
Congestive Heart Failure	5.85 (5.54-6.19)	5.94 (5.63-6.28)	0.71

eMethods. Supplementary Methods

1. All medical diagnoses made in hospitalized patients in Canada are recorded in the Canadian Institute for Health Information's Discharge Abstract Database (CIHI-DAD). The diagnoses are classified into diagnosis types based on the impact the condition had on the patient's in-hospital care. Diagnosis type M (most responsible diagnosis) refers to a condition that is the most responsible for a patient's stay in the hospital. If a patient is hospitalized for more than one condition, the one responsible for the greatest portion of the stay is selected as the most responsible.
2. Diagnoses of IHD were determined using the validated algorithm published by Tu et al.[1] This algorithm required either two physician billings within a one-year period, with one of the billings by a specialist or a family physician in a hospital or emergency room setting, or one hospitalization in the DAD (discharge abstract database). Diagnoses of AF were determined using the validated algorithm also published by Tu et al.[2], which required one entry in DAD or NACRS (national ambulatory care reporting system) with ICD-10 I48, or 4 OHIP billings on separate days within 365 days with diagnosis code 427. Diagnoses of HF were also determined using a validated algorithm involving one hospital-based diagnosis or two diagnoses in the emergency department or the ambulatory setting within 365 days of each other.

eReferences

1. Tu, K., et al., *Validation of physician billing and hospitalization data to identify patients with ischemic heart disease using data from the Electronic Medical Record Administrative data Linked Database (EMRALD).* Can J Cardiol, 2010. **26**(7): p. e225-8.
2. Tu, K., et al., *Identifying Patients With Atrial Fibrillation in Administrative Data.* Can J Cardiol, 2016. **32**(12): p. 1561-1565.

© 2026 Nakajima E et al. *JAMA Network Open*.
