# Supplementary material for: Long-Term Risk of Cardiovascular Disease After Contemporary Left-Sided Breast Radiation Therapy
Source: JAMA Netw Open. 2026 Apr 1;9(4):e264098. doi: 10.1001/jamanetworkopen.2026.4098 (PMC13044663; doi:10.1001/jamanetworkopen.2026.4098)
Supplement: Supplement 2. — Data Sharing Statement [file jamanetwopen-e264098-s002.pdf]

# Data Sharing Statement

Nakajima. Long-Term Risk of Cardiovascular Disease After Contemporary Left-Sided Breast Radiation Therapy. *JAMA Netw Open*. Published April 01, 2026.
doi:10.1001/jamanetworkopen.2026.4098

**Data**
**Data available:** No
